# SEOM clinical guidelines on nutrition in cancer patients (2018)

**DOI:** 10.1007/s12094-018-02009-3

**Published:** 2019-01-08

**Authors:** R. de las Peñas, M. Majem, J. Perez-Altozano, J. A. Virizuela, E. Cancer, P. Diz, O. Donnay, A. Hurtado, P. Jimenez-Fonseca, M. J. Ocon

**Affiliations:** 1 Medical Oncology Department, Consorcio Hospital Provincial de Castellón, Av. Doctor Clara, 19, 12002 Castellón de la Plana, Spain; 2 Medical Oncology Department, Hospital de la Santa Creu i Sant Pau, Barcelona, Spain; 3 Medical Oncology Department, Hospital Virgen de los Lirios, Alcoy, Spain; 4 Medical Oncology Department, Hospital Virgen Macarena, Sevilla, Spain; 5 Endocrinology and Nutrition Department, Hospital Universitario de Fuenlabrada, Madrid, Spain; 6 Medical Oncology Department, Hospital de León, León, Spain; 7 Medical Oncology Department, Hospital Universitario de la Princesa, Madrid, Spain; 8 Medical Oncology Department, Hospital Universitario Fundación Alcorcón, Madrid, Spain; 9 Medical Oncology Department, Hospital Universitario Central de Asturias, Oviedo, Spain; 10 Endocrinology and Nutrition Department, Hospital Clínico Universitario Lozano Blesa, Zaragoza, Spain

**Keywords:** Nutrition, Cancer, Guideline, Nutritional assessment

## Abstract

Nutritional deficiency is a common medical problem that affects 15–40% of cancer patients. It negatively impacts their quality of life and can compromise treatment completion. Oncological therapies, such as surgery, radiation therapy, and drug therapies are improving survival rates. However, all these treatments can play a role in the development of malnutrition and/or metabolic alterations in cancer patients, induced by the tumor or by its treatment. Nutritional assessment of cancer patients is necessary at the time of diagnosis and throughout treatment, so as to detect nutritional deficiencies. The Patient-Generated Subjective Global Assessment method is the most widely used tool that also evaluates nutritional requirements. In this guideline, we will review the indications of nutritional interventions as well as artificial nutrition in general and according to the type of treatment (radiotherapy, surgery, or systemic therapy), or palliative care. Likewise, pharmacological agents and pharmaconutrients will be reviewed in addition to the role of regular physical activity.

## Introduction

Disease-related malnutrition has been defined as a condition resulting from the activation of the systemic inflammatory response by an underlying disease, in this case, cancer [1, 2]. This inflammatory response causes anorexia and tissue degradation, which, in turn, can lead to significant weight loss, alterations in body composition, and decreased functional capacity [2, 3].

Specifically, cancer cachexia is a multifactorial syndrome characterized by an involuntary, sustained loss of weight and skeletal muscle mass accompanied or not by a loss of fat mass. It cannot be fully reversed by conventional nutritional support and it leads to severe functional decline. There are several stages of cancer cachexia: precachexia, cachexia, and refractory cachexia [2, 4]. Precachexia is characterized by the presence of early clinical and metabolic signs, such as anorexia and glucose intolerance that precede the loss of weight and muscle mass. The risk of progression to cachexia varies and depends on the kind of cancer, the stage of disease, the degree of systemic inflammation, the intake and response to antineoplastic therapy [6]. Refractory cachexia can result from a highly advanced or rapidly progressive cancer that does not respond to treatment. In this stage, active management of weight loss is no longer possible and life expectancy is less than 3 months [6, 7].

On the other hand, sarcopenia is a loss of muscle mass. In this case, asthenia is common; strength may be decreased, and functional capacity, limited [8]. Both cachexia [9] and sarcopenia entail a higher risk of antineoplastic treatment-related toxicity, reduced treatment response, worse surgical outcomes, and lower survival rates [8]. We must pay special attention to sarcopenic obesity, as it is an important predictor of treatment-emergent adverse events [10].

The proportion of patients who present weight loss at diagnosis is between 15 and 40%, depending on the type and stage of cancer [11]. Patients with tumors of the gastrointestinal tract, head and neck, and liver and lung cancers are at high risk for malnutrition [2]. Consequently, the incidence of malnutrition increases over the course of disease until it affects 80% of all cancer patients [12, 13]. The Spanish NUPAC study [13], designed to determine the prevalence of malnutrition in advanced cancer, confirmed a 52% rate of moderate or severe malnutrition, with a distribution of 57.7% in esophageal, 50% in gastric, and 47.1% in laryngeal cancers. More recently, a sub-analysis of the PREDYCES study revealed that 36.4% of oncology patients were at nutritional risk at the time of hospital discharge. It also demonstrated its significant association with longer hospital stays and higher healthcare costs. Despite all of this, only 1/3 of patients at nutritional risk received nutritional support [14].

It is a well-known fact that nutrition plays an important role in the prevention and treatment of cancer. The presence of malnutrition negatively affects patients’ evolution and their quality of life, increasing the incidence of infection, hospital stay, and mortality [11–13]. The aim of nutritional intervention in cancer focuses on identifying and treating malnutrition, maintaining, or improving muscle mass, as well as intervening whenever possible to address metabolic and nutritional disturbances that hinder recover and survival in these individuals [3, 4].

## Methodology

This SEOM Guideline has been developed with the consensus of ten physicians from medical oncology and endocrinology. We decided to use the US Agency for Healthcare Research and Quality Service Grading System (USPSTF) to assign a level of evidence and a grade of recommendation to the different statements contained in this guideline (Table 1) [5].

**Table 1:** Levels of evidence and grades of recommendation

Levels of evidence (I–V)	Grades of recommendation (A–E)
Evidence from at least one large randomized, controlled trial of good methodological quality (low potential for bias) or meta-analyses of well-conducted randomized trials without heterogeneity	Strong evidence for efficacy with a substantial clinical benefit, strongly recommended
Small randomized trials or large randomized trials with a suspicion of bias (lower methodological quality) or meta-analyses of such trials or of trials with demonstrated heterogeneity	Strong or moderate evidence for efficacy but with a limited clinical benefit, generally recommended
Prospective cohort studies	Insufficient evidence for efficacy or benefit does not outweigh risks or disadvantages (adverse events, costs,…), optional
Retrospective cohort studies or case–control studies	Moderate evidence against efficacy or for adverse outcome, generally not recommended
Studies without control group, case reports, expert opinions	Strong evidence against efficacy or for adverse outcome, never recommended

## General concepts

### Screening and nutritional assessment

Nutritional guidelines consistently advise screening for nutritional risk at an early stage of cancer, followed by full nutritional assessment when risk is present with the aim of establishing nutritional intervention [2]. All oncology patients should be screened at the time of diagnosis and throughout treatment using a malnutrition screening tool validated in the setting in which the tool is intended for use. Survivors should also be included in this evaluation [3, 15].

We recommend evaluating nutritional intake, weight changes, and BMI obtained either directly or by means of validated nutrition screening tools: Malnutrition Universal Screening Tool (MUST) for the outpatient clinic, Nutrition Risk Screening 2002 (NRS-2002) for inpatients, Mini Nutritional Assessment Short Form (MNA-SF) for the elderly, and the Malnutrition Screening Tool (MST) for inpatient and outpatient settings (strength of recommendation: strong; level of evidence: very low).

The Subjective Global Assessment Generated by the Patient (PG-SGA) is a tool that combines qualitative and semi-quantitative data; it is valid and reliable in identifying malnutrition as part of a comprehensive nutritional assessment in oncology patients in both ambulatory and acute care settings [3, 4]. Reduction in muscle mass can be recognized by dual X-ray absorptiometry (DEXA), computed tomography scans at lumbar level 3, or bioimpedance analysis (BIA). Physical performance can be rated using scales (ECOG, Karnofsky), dynamometry, or gait speed. Systemic inflammation can be estimated by serum C-reactive protein (CRP) and albumin [3, 15].

Nutritional assessment of food intake, muscle mass, physical performance, and systemic inflammation is recommended for all patients identified as being at risk for malnutrition by nutrition screening (strength of recommendation: strong; level of evidence: very low).

### Energy and nutritional requirements

Cancer patients have similar nutritional requirements to the healthy population, around 25–30 kcal/kg/day, with a balance between calorie intake and expenditure, including the degree of physical activity (strength of recommendation: strong; level of evidence: low).

Protein requirements are estimated to be between 1.2 and 1.5 g/kg/day. These values should be modified according to patients’ renal function, as well as any other metabolic disturbances. The contribution of water and minerals should be evaluated, especially in certain situations in which there are associated hydroelectrolyte disturbances. The administration of high-doses of vitamins and trace elements is not recommended, except in cases of established deficit [3, 15] (strength of recommendation: strong; level of evidence: moderate).

### Types of nutritional interventions

Nutritional support is indicated when there is malnutrition or risk of malnutrition; when the patient is not expected to be able to eat food for 1 week or more, or if their intake is less than 60% of their needs for more than 1–2 weeks [3]. Nutritional intervention is classified into:

- Nutritional counseling (including oral nutritional supplements) is the first and most commonly utilized intervention to manage malnourished cancer patients and a functioning gastrointestinal tract. This nutritional therapy has been shown to improve body weight, energy intake, PG-VSG scores, and certain aspects of quality of life, albeit not survival [1, 3].

Nutrition counseling (including oral nutritional supplements) should be recommended to all cancer patients who able to eat, but are malnourished or at risk for malnutrition, especially those who are undergoing oncological treatment (strength of recommendation: strong; level of evidence: moderate).

- Artificial nutrition (enteral and parenteral nutrition) is selected depending on the type of cancer, its extent, complications, treatment, and prognosis, and on the patient’s status, needs, and duration of nutritional support [3, 15].

*Enteral nutrition* by tube is indicated if intake oral is < 60% of requirement despite nutritional interventions *per os,* and gastrointestinal function is preserved. When enteral nutrition is expected to last for more than 4–6 weeks, ostomy is preferred. If there is a risk of reflux, gastroparesis, or bronchoaspiration, jejunostomy or nasojejunal tube is preferred over nasogastric or gastrostomy nutrition.

*Parenteral nutrition* is indicated when it is not possible to use the gastrointestinal tract, oral feeding and/or enteral nutrition does not suffice, and there are expectations of improvement in the patient’s quality of life and functionality with the patient’s express desire. In cases of severe intestinal insufficiency due to radiation enteritis, chronic bowel obstruction, short bowel syndrome, or peritoneal carcinomatosis, nutritional status can be maintained by parenteral nutrition. In order to prescribe home PN, the patient’s life expectancy must be more than 2–3 months and they must accept it.

We recommend enteral nutrition (EN) if oral intake remains inadequate despite nutritional counseling, and parenteral nutrition if EN is not sufficient or feasible (strength of recommendation: strong; level of evidence: very low).

### Role of physical exercise in nutritional status

Endurance exercises, aerobic training, and activities such as daily grooming or walking are considered effective strategies to enhance muscle strength and overall fitness. A systematic review reported that both aerobic and resistance exercise improves upper and lower body muscle strength more than usual care and there is some evidence that resistance exercise is perhaps more effective for improving muscle strength than aerobic exercise [3].

We recommended physical exercise in cancer patients to support or improve muscle mass and function (strength of recommendation: strong; level of evidence: high).

#### Pharmaconutrients

Pharmaconutrients are specific nutrients that have a modulating effect on the immune and metabolic function and can have beneficial effects on clinical outcomes in malnourished patients or those with advanced cancer and cachexia [16].

Some clinical studies have proven that the use of fish-derived, omega-3 polyunsaturated fatty acids (2 gr/day) in individuals with advanced cancer receiving chemotherapy improve appetite, energy zx intake, body weight, muscle mass, and/or physical activity [3, 4]. Other studies have not revealed these same results [3].

Given its clinical safety, fish oil can be suggested for malnourished patients with advanced cancer receiving chemotherapy (strength of recommendation: weak; level of evidence: low).

There is strong scientific evidence from several meta-analyses that show that enteral and oral immuno-nutrition (a combination of arginine, nucleotides, and omega-3) significantly reduce postoperative infectious complications and hospital stays in patients with cancer and upper gastrointestinal tract surgery [17].

The use of enteral immuno-nutrition in oncological patients undergoing upper gastrointestinal surgery is recommended (strength of recommendation: strong; level of evidence: high).

With the aim of increasing muscle mass, the use of certain branched-chain amino acids has been studies, such as leucine or its metabolite, Hydroxymethylbutyrate (HMB), yielding clinically inconsistent results. Likewise, there is insufficient scientific evidence to recommend the combination of glutamine-arginine-HMB [3].

When evaluating the effect of oral or parenteral glutamine on the prevention and treatment of mucositis/enteritis associated with radio/chemotherapy and on clinical outcomes in patients with hematopoietic cell transplant, results have been inconclusive [3, 4, 15, 17]. Due to the possible increased rate of tumor relapse in hematopoietic stem cell transplant patients, its use is not recommended in these subjects [3]. A systematic review has demonstrated that the administration of enteral arginine significantly reduces the incidence of fistulae and hospital stay in patients undergoing surgery for head and neck cancer, although the evidence is insufficient to recommend its use on this population [17, 18].

### Specific interventions based on cancer treatment and stage of neoplastic disease

#### Surgery

The enhanced recovery after surgery (ERAS) program seeks to lessen surgical stress, minimize catabolism, maintain nutritional status, reduce complications, and optimize recovery, making it both better and faster. The nutritional components of ERAS are: avoiding preoperative fasting, preoperative carbohydrate treatment, reestablishment of oral feeding on the first postoperative day, and early mobilization. As per this program, every patient should be screened for malnutrition and if deemed at risk, they should be provided nutritional support [17].

Management within an ERAS program is recommended for all cancer patients undergoing either curative or palliative surgery (strength of recommendation: strong; level of evidence: high).

#### Radiotherapy

Several RCTs and non-RCTs have demonstrated that individualized nutritional counseling and/or oral nutritional supplements improves nutritional intake, body weight, and QoL, enabling patients to avoid treatment interruptions and complete scheduled RT [3, 4, 19].

All patients undergoing radiation of the gastrointestinal tract or head and neck region should receive thorough nutritional assessment, individualized nutritional counseling and, if necessary, oral nutritional supplements (strength of recommendation: strong; level of evidence: moderate).

Prospective and retrospective observational trials in patients with inadequate food intake have demonstrated that enteral feeding reduces weight loss and treatment interruptions and rehospitalizations compared to oral feeding [3].

Nasogastric tube feeding or gastrostomy results in similar nutritional and clinical outcomes and overall quality of life, although the risk of tube dislodgement is lower and certain quality-of-life domains fare better with PEG, while nasogastric tubes are associated with less dysphagia and earlier weaning after completion of radiotherapy. The risks of pneumonia and other infections are similar [20, 21]. In cases in which the primary site is hypopharyngeal, the tumor is T4, or combined radio-chemotherapy, prophylactic tube feeding (either nasogastric or gastrostomy) compared to reactive tube feeding (initiated after development of dysphagia), demonstrates improvements in weight loss, quality of life, and decreases rehospitalization and treatment interruptions [22].

In severe mucositis or in obstructive tumors of the head or neck or thorax, enteral feeding is recommended using nasogastric or gastrostomy tubes (strength of recommendation: strong; level of evidence: low).

PN is ineffective and probably harmful in oncological patients in whom there is no gastrointestinal reason for intestinal failure [23]. In chronic radiation enteritis that evolves into intestinal failure, home PN appears to be a reasonable treatment option and is possibly superior to surgery [24, 25].

PN is only recommended if adequate oral/enteral nutrition is not possible (severe radiation enteritis or malabsorption) (strength of recommendation: strong; level of evidence: moderate).

### Curative or palliative pharmacological cancer treatment

Weight loss and low muscle mass prior to chemotherapy are associated with increased risk of toxicity, worse performance status, impaired quality of life, and shorter survival [3]. Targeted therapies (particularly multikinase inhibitors) have been commonly reported to result in weight loss and skeletal muscle wasting. On the other hand, weight stabilization in gastrointestinal and lung cancer patients is correlated with significant improvements in survival [3]. Dietary counseling and/or oral nutritional supplements may improve nutritional intake and quality of life, as well as stabilize body weight [3].

During anticancer drug treatment, personalized dietary counseling with oral nutritional supplements if necessary is recommended in cases of frank malnutrition and patients with decreased oral intake (strength of recommendation: strong; level of evidence: moderate).

Use of artificial nutrition (enteral or parenteral nutrition) as “routine” in all cancer patients receiving cytotoxic therapy has failed to prove a beneficial effect on survival [26]. Studies comparing EN to PN have shown that EN is feasible and possibly associated with lower complication and infection rates, as fewer cases of decreased tumor response, compared to PN [3].

Cancer patients who are malnourished or losing weight and receiving anticancer treatment who are expected to be unable to consume and/or absorb adequate nutrients for more than 1–2 weeks are candidates for artificial nutrition, preferably by the enteral route. If EN is not sufficient or possible, PN is recommended (strength of recommendation; level of evidence: very low).

### Patients with advanced cancer receiving no anticancer treatment

It is recommended that the nutritional status of patients with advanced cancer be assessed, since deficits are associated with worse quality of life and performance status [28]. Nutritional support should be carefully contemplated, taking into account the patient’s expected survival, nutritional status, potential benefit, and the expectations and wishes of both patient and their close relatives [29]. There is little evidence of benefit of nutritional support in advanced cancer patients, particularly in the last weeks of life.

We recommend that all patients with advanced cancer be routinely screened for nutritional status. However, nutritional intervention should be raised only after considering the potential benefit. In advanced, terminal phases of the disease, artificial nutrition are unlikely to provide any benefit for most patients (strength of recommendation: strong; level of evidence: low).

### Cancer survivors

As cancer survivors are at higher risk for developing second primary cancers and other chronic diseases, a diet rich in vegetables, fruits, and whole grains, and low in fats, red meats, and alcohol is recommended [3]. A review of the literature suggests that diet and exercise can have a positive impact on progressive disease and overall survival [27] (Table 2).

**Table 2:** Final recommendations

Recommendations	Strength of recommendation	Quality of evidence
Screening and nutritional assessment		
All cancer patients should be screened at the time of diagnosis and throughout treatment using a validated malnutrition screening tool	A	IV
Nutritional assessment is recommended for all patients who are identified to be at risk for malnutrition by nutrition screening	A	IV
Energy and nutricional requirements		
Cancer patients’ nutritional requirements are largely similar to those of the healthy population	B	III
Proteins, water, and minerals requirements should be evaluated especially in certain situations. The administration of high-doses of vitamins and trace elements is not recommended	B	III
Types of nutritional interventions		
Nutrition counseling should be recommended to all cancer patients who able to eat, but are malnourished or at risk for malnutrition	B	III
Enteral nutrition, if oral intake remains inadequate despite nutritional counseling, and parenteral nutrition, if enteral nutrition is not sufficient or feasible	B	V
Role of physical exercise in nutritional status		
Physical exercise in cancer patients to support or improve muscle mass and function	A	II
Pharmaconutrients		
The use of fish oil in malnourished patients with advanced cancer receiving chemotherapy	C	IV
The use of enteral immuno-nutrition in cancer patients undergoing upper gastrointestinal surgery	A	II
Interventions relevant to specific patients categories		
Management within an ERAS program is recommended for all cancer patients undergoing either curative or palliative surgery	A	II
Nutritional assessment, individualized nutritional counseling, and, if necessary, oral nutritional supplements in all patients undergoing radiation of the gastrointestinal tract or of the head and neck	B	III
In severe mucositis or in obstructive tumors of the head-neck or thorax, enteral feeding is recommended using nasogastric or gastrostomy tubes	B	IV
Parenteral nutrition is recommended if adequate oral/enteral nutrition is not possible (severe radiation enteritis or malabsorption)	B	III
During anticancer drug treatment, personalized dietary counseling, with oral nutritional supplements if necessary, is recommended in cases of frank malnutrition and patients with decreased oral intake	B	III
Malnourished cancer patients receiving anticancer treatment who are expected to be unable to ingest and/or absorb adequate nutrients for more than 1–2 weeks are candidates for artificial nutrition (enteral or parenteral)	B	V
In advanced terminal phases of the disease, artificial nutrition is unlikely to provide any benefit for most patients	B	IV
In cancer survivors: maintaining a BMI between 18.5 and 25 kg/m^2^, physical activity, and a healthy diet	B	IV

We recommend that a BMI of between 18.5 and 25 kg/m^2^ be maintained, in addition to physical activity and a healthy diet (strength of recommendation: strong; level of evidence: low).
